# Editorial: Progression of diabetic kidney disease

**DOI:** 10.3389/fneph.2024.1439950

**Published:** 2024-07-26

**Authors:** Mariana Charleaux de Ponte, Larissa de Araújo

**Affiliations:** ^1^ Laboratory of Cellular and Molecular Bases of Renal Physiology, Department of Physiology and Biophysics, Institute of Biomedical Sciences, University of Sao Paulo, Sao Paulo, Brazil; ^2^ Laboratory of Renal Physiology, Department of Physiology and Biophysics, Institute of Biomedical Sciences, University of Sao Paulo, Sao Paulo, Brazil

**Keywords:** diabetic kidney disease, albuminuria, cannabinoid type-1 receptor, oxygen stress inducible enzyme, SGLT2 inhibitors

Diabetes mellitus is a metabolic syndrome characterized by sustained hyperglycemia. It is primarily classified into two etiopathogenetic categories: type 1 (T1DM) and type 2 (T2DM) diabetes. T2DM is associated with insulin resistance, leading to decreased glucose uptake by tissues. Between 30 to 40% of individuals diagnosed with either T1DM or T2DM develop diabetic kidney disease (DKD). DKD first manifests as microalbuminuria and can progress to macroalbuminuria accompanied by progressive kidney function loss. Glomerular injury, driven by hyperglycemia, results in albumin excretion. It is known that despite glycemic control, some patients still develop DKD, indicating that other mechanisms are involved in the progression of renal function loss. This Research Topic was created to provide current data and reviews addressing the mechanisms of DKD progression and possible therapeutic approaches.

Sigfrids and Groop presented a review with central findings on the incidence, progression, and regression of T1DM from 1980 to 2020 based on albuminuria. Albumin detection became accessible in the 80s, and at that time, studies were still very imprecise. The authors observed diabetic patients for an average of 21.8 years from diagnosis. This study showed that the incidence of albuminuria increased within the first 10 years of diabetes and remained stable until 25 years, when it started to decrease. Studies have also focused on the temporal trend of albuminuria in T1DM. A significant reduction in the incidence of severe albuminuria over time was observed. It was described that this reduction occurred between the 70s and 80s, but from the 90s onwards, this was no longer observed. This suggests a delay in severe albuminuria in diabetes in the modern era. Regarding progression, some studies show that glycemic treatment is efficient in reducing the progression of albuminuria, but others state that there is still concern about the association of albuminuria with a greater risk of vascular complications. Recent studies show that albuminuria can even regress over time using inhibitors, such as SGLT2 and the renin-angiotensin system. The mechanisms underlying this response include glycemic and blood pressure control, as well as a decrease in triglyceride levels. However, the extent to which albuminuria regression is related to changes in the risk of adverse vascular outcomes and mortality still differs between studies. In T2DM, there is a reduction in cardiovascular risks after the regression of albuminuria, while in T1DM, this association has not yet been established. Studies also show a decreased incidence of kidney failure over time. However, there are some geographic divergences, and therefore, DKD should still be highlighted in research, especially considering these regional differences.

Jacquot et al. showed an important mechanism associated with the progression of DKD in T1DM. It was demonstrated that a novel peripherally restricted cannabinoid type-1 receptor (CB1R) inverse agonist has a potential therapeutic role in the progression of diabetic nephropathy induced by streptozotocin (STZ) treatment. CB1R is mostly expressed in the proximal tubular cells and podocytes, which present serious dysfunctions in DKD, a condition that leads to albuminuria. The first commercially available CB1R inverse agonist, rimonabant, had already shown beneficial effects on glycemic control in obese and/or diabetic patients. Still, due to serious side effects on the central nervous system (CNS), the drug was discontinued. Specific peripheral agonists that did not penetrate the CNS did not show the same effects as rimonabant. Thus, the authors decided to investigate the effects of a new peripheral CB1R inverse agonist INV-202 (0.3 mg/kg and 3 mg/kg) in STZ-induced T1DM. INV-202 decreased albumin excretion, improved glomerular and tubular function, reduced fibrosis, and the expression of oxidative stress and inflammation markers. The dose of 3 mg/kg was more efficient than the dose of 0.3 mg/kg. Therefore, this study showed that INV-202 has therapeutic potential in reducing the progression of kidney disease after treatment with STZ, indicating that the CB1R pathway is involved in diabetic nephropathy.

Zhai et al. also showed an important pathway for the progression of DKD, associated with the oxygen stress-inducible enzyme (HO-1). This enzyme promotes the generation of antioxidants and anti-apoptotic molecules essential for cellular protection or post-injury regeneration processes. The role of HO-1 in delaying renal progression in several models of acute kidney injury has been reported, such as those induced by ischemia/reperfusion injury, cisplatin nephrotoxicity, or ureteral obstruction. In the case of DKD, HO-1 inhibition increased the number of apoptotic cells and albumin permeability in renal tissue in T1DM. The important role of HO-1 in increasing insulin sensitivity and reducing adipose tissue in T2DM has also been shown. In addition, preclinical studies demonstrated that HO-1 could be used as an early biomarker of DKD. Thus, the modulation of this enzyme has a relevant role in the progression or delay of DKD in both T1DM and T2DM.

It is known that SGLT2 inhibitors are proving to be an important tool for improving kidney function in diabetes. Wang et al. showed that these inhibitors are also efficient for the treatment of type 2 cardiorenal syndrome, a condition in which heart failure leads to renal dysfunction with potential progression to chronic kidney disease. SGLT2 inhibitors improve renal function by decreasing renal tubular congestion (through increased natriuretic response) and improving the metabolism of proximal tubule cells. These inhibitors also act to reduce the activation of inflammatory, fibrotic, and oxidative stress pathways. Therefore, this review aligns with other studies concluding the beneficial effects of SGLT2 inhibition in kidney dysfunction.

In conclusion, this topic has presented some important insights into the incidence, progression, and regression of albuminuria in DKD. Furthermore, some mechanisms underlying the progression and regression of DKD were demonstrated, such as those triggered by CB1R and the HO-1 enzyme, respectively. It was also possible to observe an important therapeutic role of SGLT2 inhibitors, commonly used in cases of diabetes, in type 2 cardiorenal syndrome.

## Author contributions

MdP: Writing – original draft, Writing – review & editing. LdA: Writing – original draft, Writing – review & editing.

